# Cauda equina syndrome following an uneventful spinal anesthesia in a patient undergoing drainage of the Bartholin abscess: A case report

**DOI:** 10.1097/MD.0000000000010693

**Published:** 2018-05-11

**Authors:** Waldo Merino-Urrutia, Milca Villagrán-Schmidt, Priscilla Ulloa-Vásquez, Rubén Carrasco-Moyano, Alberto Uribe, Nicoleta Stoicea, Sergio D. Bergese

**Affiliations:** a Department of Surgery, Orthopedic and Anesthesiology, Universidad de La Frontera, Hospital Hernán Henríquez Aravena, Temuco, Chile; b Department of Anesthesiology; c Department of Neurological Surgery, The Ohio State University Wexner Medical Center, Columbus, Ohio.

**Keywords:** cauda equina syndrome, neurotoxic, spinal anesthesia

## Abstract

**Rationale:**

Neuraxial anesthesia is a commonly used type of regional anesthesia. Cauda equina syndrome is an unusual and severe complication of neuraxial anesthesia, and is caused by damage to the sacral roots of the neural canal. We present a case of cauda equina syndrome following spinal anesthesia in a patient who underwent Bartholin abscess drainage.

**Patient concerns:**

A 23-year old female scheduled to undergo surgical drainage of Bartholin abscess. Spinal anesthesia was performed with bupivacaine and fentanyl. There were no perioperative adverse events reported. On postoperative day 1, the patient went to the emergency department describing bilateral weakness and pain of the lower extremities (LE).

**Diagnoses:**

Lumbar magnetic resonance imaging showed increased gadolinium accumulation in the neural sheath at the level of the cauda equina tracts, consistent with the diagnosis of arachnoiditis and the diagnosis of cauda equina was established.

**Interventions:**

The patient received the following emergent treatment: 75 mg pregabalin (oral) every 12 hours, 20 mg (8 drops) tramadol (oral) every 8 hours, and 4 mg dexamethasone (intravenous) every 6 hours. On postoperative day 4, the patient still experienced bilateral flaccid paraparesis (accentuated in the left side), neuropathic pain in low extremities, and left brachial monoparesis. Hence, dexamethasone was instantly replaced with 1 g methylprednisolone (intravenous) for 5 days.

**Outcomes:**

After completing 5 days of methylprednisolone, on postoperative day 9, the patient experienced less pain in left extremities, osteotendinous reflexes were slightly diminished, and she was able to walk with difficulty for 3 to 5 minutes. Greater mobility was evidenced, with right proximal and distal low extremities Medical Research Council Scale grades of 2 and 3 and left proximal and distal low extremities Medical Research Council Scale grades 1 and 2, respectively. Oral prednisone was restarted. Consequently, she was discharged home in stable conditions on postoperative day 25 with a prescription for sertraline, clonazepam, pregabalin, paracetamol, and prednisone.

**Lesson:**

The early detection and treatment of complications after neuraxial anesthesia is essential to minimize the risk of permanent damage.

## 1 Introduction

Neuraxial anesthesia (NA) is a commonly used type of regional anesthesia employing the injection of local anesthetics along with other additives into fat tissue surrounding the nerve roots (epidural anesthesia [EA]) or into the intrathecal space containing the cerebrospinal fluid (CSF) surrounding the spinal cord (spinal anesthesia [SA]).^[1–3]^ NA is described as a safe, efficient, and cost-effective technique that provides appropriate perioperative analgesia and is frequently used in surgical procedures performed in the lower abdominal and pelvic region, lower extremities (LE), and cesarean section.^[2,3]^ NA has been linked to the reduction of cardiopulmonary complications, postoperative mortality, and deep vein thrombosis prophylaxis.^[4]^ The occurrence of neurologic complications after NA is very rare—between 1–1000/1–100,000^[4]^; nevertheless, anesthesia care providers should be always aware of its possible postoperative neurologic complications.

Cauda equina syndrome (CES) is an unusual and severe complication of NA induced by damage to the sacral roots of the neural canal.^[2,5,6]^ The etiology of CES is diverse, including but not limited to direct or indirect trauma after several puncture attempts, infection, ischemia or compression of spinal cord or nerve roots by a hematoma. Certain drugs can display direct neurotoxicity when administered intrathecally, triggering inflammatory reaction.^[2,5,7]^ The paucity of literature concerning this matter made the management tailored on etiology and surgical decompression, or conservative treatment based on signs and symptoms.^[2,5,7]^

The syndrome is characterized by proximal weakness of LE, loss of sensitivity, lower back pain, and sciatica that can lead to different grades of sexual dysfunction and intestinal and/or vesical sphincter dysfunction, perineal numbness, and even paraplegias.^[2,8]^

We report a case of cauda equina syndrome following SA using Bupivacaine 0.75% in a woman patient who underwent Bartholin abscess drainage.

## 2 Case presentation

A 23-year-old woman, 80  kg, 1.56 m (body mass index: 32.87 kg/m^2^), American Society of Anesthesiologist physical status (ASA) II, with no relevant history of neurological pathologies or surgical history, and allergic to metamizole was admitted to the hospital and scheduled to undergo surgical drainage of Bartholin abscess. Vital signs, family and medical history, physical examination, and laboratory tests were unremarkable. Per standard procedures, a verbal consent was obtained from the patient in order to publish the case report with de-identified data. Subsequently, for a proper documentation, a written informed consent was obtained as well.

Prior to spinal anesthesia procedure, the lumbar area was prepared with ethanol 70% (diethyl phthalate 0.2%). Spinal anesthesia was performed with the patient in a sitting position, and only 1 puncture attempt was performed using a 25-G Whitacre spinal needle (Uniever; Unisys Corp, Tokyo, Japan) using a 25-G spinal introducer at the L3–L4 interspace. After a few drops of clear CSF was confirmed, 7.5 mg of hyperbaric bupivacaine 0.75% (Bupivan, Hospira) with 15 μg of fentanyl (Sanderson) was intrathecally administered at the L3–L4 interspeace. There were no adverse events reported during introducer placement, insertion of the needle, or during intrathecal medication administration.

The surgical drainage of the Bartholin abscess was performed in a lithotomy position and was uneventful. The length of the surgical procedure was 25 minutes, and following surgery the patient was transferred to the post anesthesia care unit (PACU). During her PACU stay (2 hours), the patient remained hemodynamically stable, and once considered completely recovered from the motor block, was transferred to the gynecology ward. Consequently, the patient was discharged home in stable condition 12 hours after surgery.

**Figure 1:**
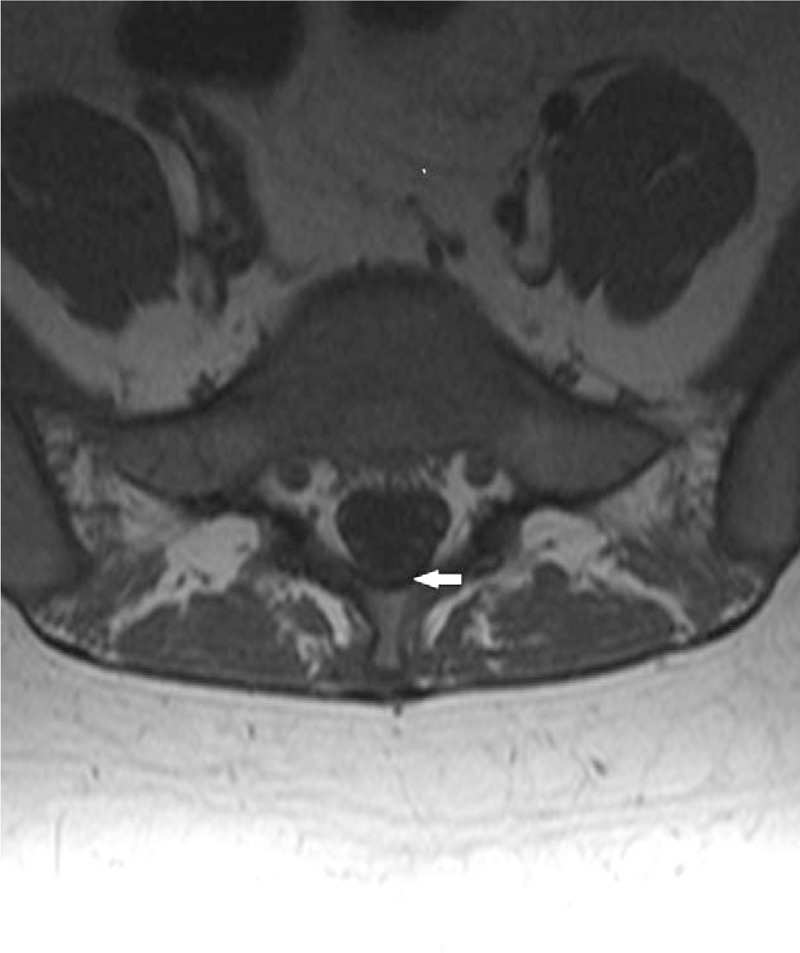
Axial sequence with gadolinium. Higher gadolinium captured in the cauda equina tracts.

**Figure 2:**
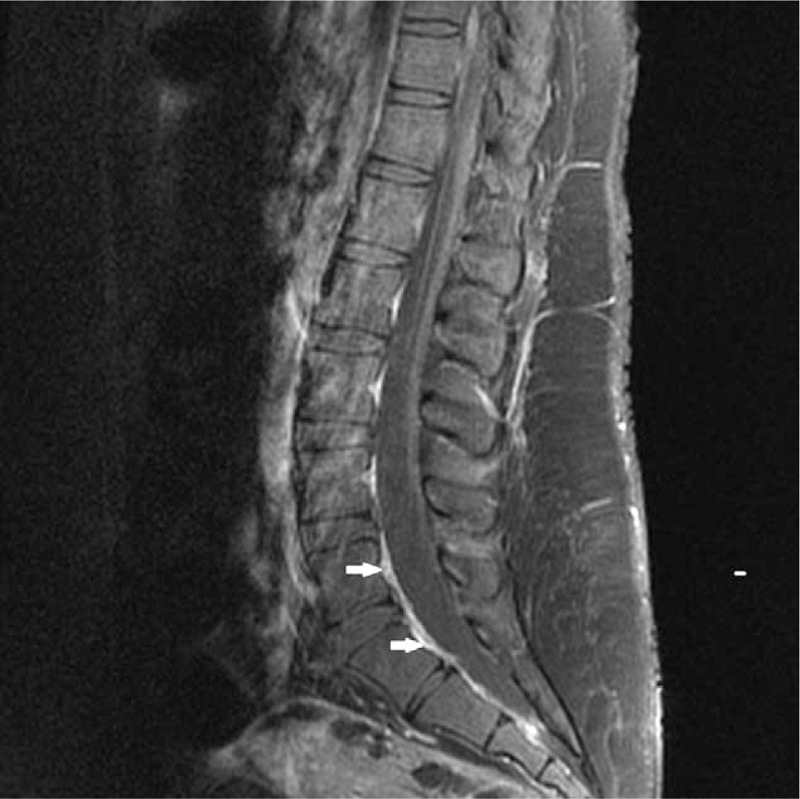
Sagittal sequence with gadolinium. Dural enhancement.

On the morning of postoperative day 1, the patient went to the emergency department describing bilateral weakness and pain in LE. A neurology consult was immediately requested, and the neurology assessment revealed a Glasgow Coma Scale (GCS) of 15, intact cranial nerves, left brachial monoparesis, left crural paresthesia, and paresis (Medical Research Council [MRC] Scale grade 2,^[5,9]^ right crural paresis [MRC Scale grade 3^[5,9]^]), hypoesthesia in both legs with sensitivity loss below level T9, decreased osteotendinous reflexes (OTR), bilateral sole flexor reflexes, and urinary retention. A computerized axial tomography (CAT) scan without contrast revealed no evidence of brain and spinal lesions. Lumbar magnetic resonance imaging (MRI) showed increased gadolinium accumulation in the neural sheath at the level of the cauda equina tracts, consistent with the diagnosis of arachnoiditis (Figs. 1 and 2).

Based on the aforementioned information, the diagnosis of CES was established and the patient received the following emergent treatment: 75 mg pregabalin (oral) every 12 hours, 20 mg (8 drops) tramadol (oral) every 8 hours, and 4 mg dexamethasone (intravenous) every 6 hours. Cell blood count and differential, C-reactive protein, urine test, plasma electrolytes, creatine kinase-MB, creatinine kinase test, and liver function panel tests were all normal.

On postoperative day 4, the patient still experienced bilateral flaccid paraparesis (accentuated in the left side), neuropathic pain in LE, and left brachial monoparesis. Hence, dexamethasone was instantly replaced with 1 g methylprednisolone (intravenous) for 5 days. On postoperative day 5, the neuropathic pain in LE was diminished and a multidisciplinary approach with a physiatrist, psychologist, physiotherapist, and occupational therapist was started. The patient recovered sphincter control and could move her left toes and sit with stable trunk control, but the bilateral paresthesia and pain in the left LE extremity persisted.

After completing 5 days of methylprednisolone, on postoperative day 9, the patient experienced less pain in left LE, OTR were slightly diminished, and she was able to walk with difficulty for 3 to 5 minutes. Greater mobility was evidenced, with right proximal and distal LE MRC Scale grades of 2 and 3 and left proximal and distal LE MRC Scale grades 1 and 2, respectively. Oral prednisone was restarted.

Consequently, she was discharged home in stable conditions on postoperative day 25 with a prescription for sertraline, clonazepam, pregabalin, paracetamol, and prednisone and referred for a neurorehabilitation program.

Fortunately, the weakness of the bilateral LE gradually continued to improve over time and, during the last regular outpatient follow-up performed 2 months after this event, regression of the signs and symptoms were evidenced, OTR were normal, and the patient was able to move all her extremities normally and walk without any support. She only noted a persistent light pain in the left thigh, so analgesics and physical therapy were maintained.

## 3 Discussion

Neuraxial anesthesia is known to be associated with various complications, including cauda equina syndrome.^[10]^ This condition is characterized by weakness and loss of sensitivity in the LE, sexual dysfunction, intestinal and/or vesical sphincter dysfunction, and uni- or bilateral pain in the gluteal region.^[5,8]^ The clinical manifestation of this syndrome is unusual, with an approximate frequency of permanent neurological damage between 0.3 and 1.2 per 100,000 spinal anesthesia administrations.^[4]^

The etiologies of cauda equina syndrome include the lithotomy position, direct or indirect trauma of the spinal cord, compression or ischemia of the spinal cord, neurotoxicity caused by local anesthetics, and some iatrogenic causes such as: manipulation, contamination of local anesthetics with chemical substances, and postoperative complications like hematomas.^[5,8]^

In this case, the syndrome appeared to be related to spinal anesthesia with hyperbaric bupivacaine 0.75%, known to be less frequently associated with CES than lidocaine use. Higher concentration of lidocaine use may result in higher neurotoxicity, as lidocaine has been seen to have the greatest neurotoxicity of all local anesthetics in use. In addition, some authors reported that persistent functional impairment occurred only after intrathecal lidocaine.^[8]^ Other studies have reported that dibucaine has the highest risk of neurotoxicity, followed by tetracaine, lidocaine, bupivacaine, then ropivacaine.^[11]^ Interestingly, one notes that the use of dextrose in hyperbaric solutions does not appear to have a harmful effect on the nerve roots.^[12]^

Another factor proven to increase the risk of cauda equina syndrome is the lithotomy position, since it favors the accumulation of anesthetic in the sacral area and the relaxation of the nerve roots and vasa nervorum.^[12]^ However, in this case, it is worth mentioning that the patient was placed in lithotomy position in the operating room for a short period of time (length of surgery = 25 minutes).

The patient's obesity is another favoring factor, and early perambulation might also be a relevant factor in this case, since the patient was discharged 12 hours after the surgical procedure.^[12]^

## 4 Conclusion

In conclusion, we report a case of cauda equina syndrome after uneventful single spinal administration of bupivacaine 0.75%. The explanation for this complication is uncertain, but could be due to a combination of needle or introducer trauma at the time of insertion or due to the neurotoxic properties of Bupivacaine. We emphasize that the early detection and treatment of complications after NA is essential to minimize the risk of permanent damage.

## Author contributions

**Conceptualization:** Waldo Merino-Urrutia, Milca Villagrán-Schmidt, Priscilla Ulloa-Vásquez, Ruben Carrasco-Moyano.

**Data curation:** Alberto Uribe, Waldo Merino-Urrutia, Milca Villagrán-Schmidt, Priscilla Ulloa-Vásquez, Ruben Carrasco-Moyano.

**Formal analysis:** Alberto Uribe.

**Investigation:** Alberto Uribe, Waldo Merino-Urrutia, Ruben Carrasco-Moyano.

**Methodology:** Alberto Uribe, Waldo Merino-Urrutia, Ruben Carrasco-Moyano.

**Project administration:** Ruben Carrasco-Moyano.

**Supervision:** Alberto Uribe, Waldo Merino-Urrutia, Ruben Carrasco-Moyano, Nicoleta Stoicea, Sergio D. Bergese.

**Validation:** Waldo Merino-Urrutia.

**Visualization:** Alberto Uribe.

**Writing – original draft:** Alberto Uribe, Waldo Merino-Urrutia, Milca Villagrán-Schmidt, Priscilla Ulloa-Vásquez, Ruben Carrasco-Moyano, Nicoleta Stoicea, Sergio D. Bergese.

**Writing – review & editing:** Alberto Uribe, Waldo Merino-Urrutia, Milca Villagrán-Schmidt, Priscilla Ulloa-Vásquez, Ruben Carrasco-Moyano, Nicoleta Stoicea, Sergio D. Bergese.
